# Lichen Planus Follicularis Tumidus of the Vulva: A Case Report and Literature Review

**DOI:** 10.1155/crip/9945177

**Published:** 2026-07-09

**Authors:** Kong Xiangjun, Shao Lili, Hou Shuping, Zhao Qian, Wang Huiping

**Affiliations:** ^1^ Department of Dermatology and Venereology, Tianjin Institute of Sexually Transmitted Diseases, Tianjin Medical University General Hospital, Tianjin, China, tjmugh.com.cn; ^2^ Department of Dermatology and Venereology, Tianjin Medical University General Hospital, Tianjin, China, tjmugh.com.cn

**Keywords:** cutaneous, dermatopathology, lichen planus, LPFT, vulva

## Abstract

Lichen planus follicularis tumidus (LPFT) is a rare variant of follicular lichen planus that predominantly affects the postauricular region, although cases involving the nasal ala and vulva have also been reported. The distinctive histopathology of LPFT can lead to diagnostic confusion with plaque parapsoriasis, lichenoid keratosis, and folliculotropic mycosis fungoides. We herein report a case of LPFT in a 54‐year‐old woman presenting with a vulvar lesion that exhibited plaque‐like and verrucous features. Histopathologically, the lesion demonstrated typical lichenoid inflammation that surrounded dilated follicular infundibula. The patient achieved complete resolution following surgical excision.

## 1. Introduction

Lichen planus comprises various subtypes that affect the skin, oral mucosa, and genital mucosa. Follicular lichen planus is an uncommon subtype, with lichen planus follicularis tumidus (LPFT) representing a rare variant. It was first described by Belaïch et al. [1], and is considered a variant of lichen planus. Earlier reports have depicted LPFT as predominantly affecting women, and it commonly involves the postauricular and facial regions; however, authors have recently described vulvar involvement [1]. We herein describe a case of LPFT affecting the mons pubis, clinically presenting as a plaque‐like and verrucous lesion.

### 1.1. Case Presentation

A 54‐year‐old woman presented with a 5‐year history of vulvar lesions. Five years previously, she noted the spontaneous appearance of a pea‐sized, exophytic lesion on the mons pubis and without associated pain or pruritus, ulceration, or exudate, with the lesion gradually increasing in size. The patient′s medical history included nonsmall cell lung cancer and thyroid cancer that were both diagnosed years ago and treated successfully with early surgical intervention, without recurrence on recent follow‐up.

**Figure 1:**
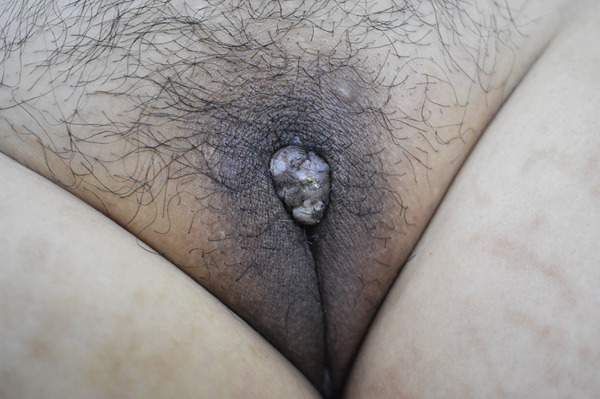
Verrucous follicular lichen planus of the vulva.

**Figure 2:**
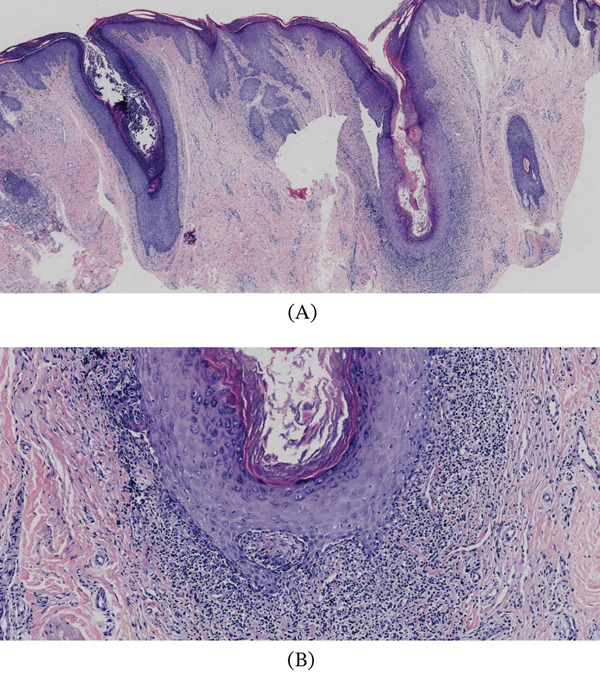
(A) Markedly dilated follicular infundibula with irregular epidermal thickening between adjacent follicles. (H&E; original magnification, ×20) and (B) dilated follicular infundibula filled with parakeratotic material. We observed wedge‐shaped acanthosis of the follicular wall, liquefactive degeneration of the basal layer, and dense lichenoid lymphocytic infiltrate surrounding the follicular infundibula (H&E; original magnification, ×100).

**Figure 3:**
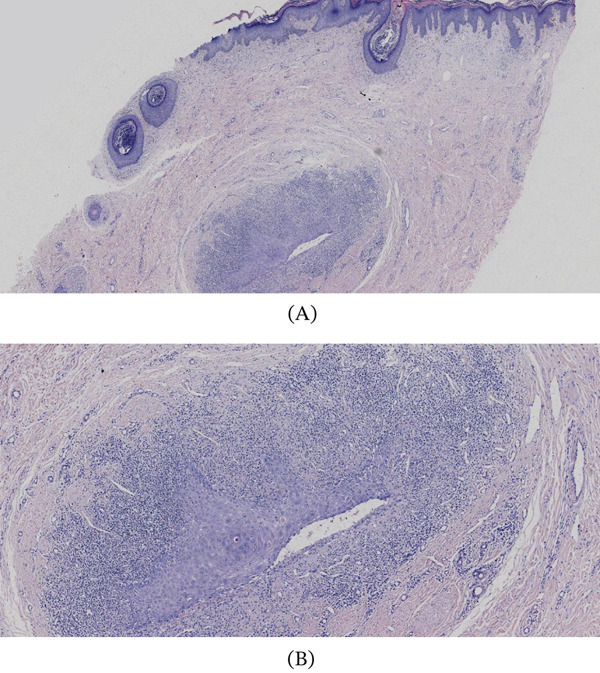
(A) Keratin cysts within the reticular dermis. (H&E; original magnification, ×20). (B) Some cyst walls showed liquefactive degeneration; and we observed prominent lichenoid inflammatory infiltrate with scattered keratinocyte necrosis (H&E; original magnification, ×50). Two biopsies of the verrucous plaque revealed dilated follicular infundibula filled with parakeratotic material. The interfollicular epidermis appeared unremarkable, although melanophages were present in the superficial dermis (Figure 2). Wedge‐shaped acanthosis of the follicular wall, keratinocyte necrosis, liquefactive degeneration of the basal layer, and a dense lichenoid lymphocytic infiltrate with a few plasma cells were noted (Figure 3). Periodic acid–Schiff (PAS) staining was negative for fungi.

Dermatologic examination revealed no lesions on the trunk, extremities, oral mucosa, or vaginal mucosa. We observed a 1.5‐cm exophytic, grayish‐white, rough, and irregularly surfaced lesion with comedones on the mons pubis (Figures 1, 2, and 3).

Due to the often‐refractory nature of LPFT to various treatment modalities, complete surgical excision of the verrucous plaque was performed, and the patient remained disease‐free at her 1‐year follow‐up.

## 2. Discussion

LPFT is a rare variant of lichen planus that is characterized by lichenoid inflammation that affects hair follicles and clinically presents as tumid plaques. Only a few cases have been reported in the extant literature (Table 1).

**Table 1:** Review of reported cases of lichen planus follicularis tumidus (LPFT).

Reference	Patient (age/sex) and PMHx	Morphology and location	Histopathologic findings	Treatment
Termin et al. [2]	59 F; T2DM, hypothyroidism, HLD, HTN, VTE, Crohn′s	Groin/labia majora: boils, milia‐like cysts	Lichenoid interface dermatitis; follicular plugging	IL triamcinolone; oral prednisone
Wankhade et al. [3]	62 M; HTN, DM	Retroauricular: violaceous tumid plaque with milia‐like cysts/comedones	Dense perifollicular lymphocytic infiltrate; keratin‐filled cysts	Oral prednisolone
Jiménez‐Gallo et al. [4]	50 F; Hx of LP, Hep B/C	Face: wart‐like lesions	Lichenoid infiltrate surrounding follicles/cysts; colloid bodies	Cyclosporine
Saggini et al. [5]	63 M; none	Nose: erythematous‐brownish plaque	Dense lymphohistiocytic infiltrate around dilated follicles	NR
Hassab‐El‐Naby et al. [6]	63 M; HCV	Nasal ala: coalesced skin‐colored nodules	Dense band‐like lymphocytic infiltrate; dilated infundibula; dyskeratotic cells	IL triamcinolone
Chau et al. [7]	67 M; SCC (Hx)	Retroauricular: lichenified hyperpigmented nodule	Dense lichenoid infiltrate centered around cystically dilated follicles	NR
Ozden et al. [8]	54 F; Hashimoto′s	Retroauricular: pruritic dark‐brown lesion	Band‐like lymphohistiocytic infiltrate; pigmentary incontinence	Clobetasol 0.05% cream
Vázquez et al. [9]	48 M; none	Scalp/retroauricular/sacral: violaceous infiltrative plaques	Dense lichenoid band‐like infiltrate; large keratin‐filled follicular cysts	NR
Vázquez et al. [9]	76 F; none	Temporo‐frontal: whitish/yellowish plaque	Dense lichenoid band‐like infiltrate; large keratin‐filled follicular cysts	NR
Rongioletti et al. [10]	57 F; none	Retroauricular/chin/cheek: erythematous/edematous plaques	Dense lichenoid band‐like lymphocytic infiltrate; follicular dilation	Isotretinoin
Kaunitz and Kim [11]	50 M; melanoma (Stage IV)	Malar/preauricular: cystic papules/plaques	Dense lymphohistiocytic infiltrate; interface changes; pseudoepitheliomatous hyperplasia	Topical/IL corticosteroids
Our case	54 F; lung and thyroid CA	Mons pubis: wart‐like patch with milia‐like cysts/comedones	Dense lichenoid lymphocytic infiltrate; dilated keratin‐filled infundibula	Surgical resection

Abbreviations: CA, cancer; DM, diabetes mellitus; HLD, hyperlipidemia; HTN, hypertension; IL, intralesional; LP, lichen planus; NR, not reported; PMHx, past medical history; SCC, squamous cell carcinoma; T2DM, Type 2 diabetes mellitus; VTE, venous thromboembolism.

In this literature review, we systematically searched relevant medical databases for clinical case reports with a definitive diagnosis of LPFT. A total of 12 cases were included in this analysis, which incorporates the case presented in this report to ensure the comprehensiveness and accuracy of the data. Cases were selected based on the availability of sufficient clinical and histopathologic documentation; studies lacking detailed descriptions or clear diagnostic criteria were excluded.

The review includes a total of 12 cases. The gender distribution shows a slight male predominance, consisting of seven males and five females. The lesions were most commonly localized to the head and neck region (particularly the retroauricular area, nose, and cheeks), though occurrences were also noted in the anogenital area (groin, labia majora, and clitoris) and the lumbosacral zone. Among the 12 reviewed cases, only one occurred in the vulvar region. Although that case involved the labia majora, our patient′s lesion was uniquely located on the mons pubis, highlighting the rare involvement of the pubic region in LPFT. Notably, compared with our case, that specific patient exhibited more classic histopathological features of lichen planus, characterized by a dense lichenoid lymphocytic infiltrate in the superficial dermis. However, clinical features such as milia‐like cysts and comedones were not prominent in that instance.

The histopathology of LPFT is characterized by a distinctive constellation of follicular‐centric changes. Key clinical features, as reported in the literature, reveal that LPFT typically presents as pigmented papulo‐plaque lesions accompanied by milia‐like cysts and comedones [2, 3, 9, 10]. The histopathology of LPFT is characterized by a distinctive constellation of follicular‐centric changes. The primary diagnostic features include a dense, band‐like lichenoid lymphocytic infiltrate that characteristically surrounds and targets the follicular infundibula. These infundibula exhibit marked cystic dilation and are typically packed with keratin, often extending deeply into the reticular dermis. Associated features of a severe lichenoid interface dermatitis are frequently present, including significant pigmentary incontinence and the formation of colloid bodies; however, epidermal inflammation remains relatively mild, primarily manifesting as acanthosis. Crucially, the absence of two specific markers—substantial mucin deposition and lymphocytic atypia—serves as the primary evidence to distinguish this condition from its main histopathologic mimics.

The identification of LPFT relies upon the synthesis of its unique follicular‐centric inflammatory pattern and deep‐seated keratin‐filled cysts. To systematically differentiate LPFT from its primary mimics, the following distinctions are observed: (1) Folliculotropic mycosis fungoides (FMF): The lack of lymphocytic atypia in LPFT provides the most critical feature to exclude FMF, which is defined by the presence of atypical lymphocytes and epidermotropism. (2) Lichen planopilaris (LPP), discoid lupus erythematosus (DLE), and nevus comedonicus: Unlike LPP and DLE, which frequently demonstrate significant mucin deposition or less pronounced infundibular cystic changes, LPFT is characterized by a more prominent, deep‐seated distribution of keratin‐filled follicular infundibula combined with a dense, lichenoid lymphocytic infiltrate. Nevus comedonicus is readily distinguished by the absence of an intense, lichenoid interface dermatitis. (3) Milia en plaque: Although both conditions may involve dermal cysts, LPFT is distinguished by the presence of a dense, lichenoid lymphocytic infiltrate surrounding the follicular infundibula, a feature typically lacking in milia en plaque. (4) Keratosis lichenoides chronica (Nekam′s disease): Although this condition may present with lichenoid inflammation, it is clinically manifested by multiple verrucous lesions and histologically lacks the follicular‐centric inflammatory changes that define LPFT.

Clinically, LPFT may mimic conditions such as proliferative pemphigus vulgaris and verrucous cutaneous tuberculosis, similarly exhibiting a predisposition to intertriginous, compressed, and friction‐prone sites. The proliferative changes in intertriginous dermatoses, exemplified by pemphigus vegetans, have been hypothesized to represent a protective phenomenon secondary to local factors such as occlusion and maceration [12]. By analogy, it is plausible to speculate that the verrucous component of LPFT may arise from similar underlying mechanisms; however, this remains a postulation that necessitates further empirical investigation. Furthermore, the high density of sebaceous and apocrine glands in these intertriginous regions could potentially create a moist, macerated microenvironment that might facilitate secondary microbial proliferation, though this remains to be validated in future studies [5].

Although the reported comorbidities in previous reports include viral infections such as hepatitis C virus infection, infection was inactive in two cases [4, 6]. Autoimmune diseases—including Hashimoto′s thyroiditis [8] and Crohn′s disease [1]—have also been reported to be associated with LPFT. Vulvar erosive lichen planus also often demonstrates a high rate (38%) of associated autoimmune disease. Although our patient exhibited a history of nonsmall cell lung cancer and thyroid cancer, both were successfully treated with early surgical resection without recurrence on follow‐up; one reported case, however, manifested a squamous cell carcinoma (SCC) within the LPFT lesion. However, a review of previous pathology reports suggested that this diagnosis may have been confounded by acanthosis within the LPFT lesion; and therefore, a direct causal link between LPFT and malignancy remains inconclusive [7]. Concurrently, a link exists between LPFT and the use of pharmacotherapy, with one reported case of LPFT triggered by PD‐1 blockade used for melanoma treatment [11].

Treatment for LPFT often involves oral corticosteroids (prednisone) and intralesional corticosteroids (triamcinolone). However, treatment response is often slow, with persistent comedones and milia after plaque resolution. Cyclosporine may be considered if corticosteroids are ineffective. For highly localized lesions, surgical excision may be the optimal therapeutic approach, as keratin‐filled comedones and cysts may interfere with the efficacy of anti‐inflammatory therapies. In our case, the lesion was successfully resolved through surgical resection, suggesting that this approach may be safer for elderly patients than prolonged courses of oral corticosteroids.

## Funding

No funding was received for this manuscript.

## Conflicts of Interest

The authors declare no conflicts of interest.

## Supporting information


**Supporting Information** Additional supporting information can be found online in the Supporting Information section.

## Data Availability

The data that support the findings of this study are available from the corresponding author upon reasonable request.
