# Supplementary material for: Lichen Planus Follicularis Tumidus of the Vulva: A Case Report and Literature Review
Source: Case Rep Pathol. 2026 Jul 9;2026:9945177. doi: 10.1155/crip/9945177 (PMC13347628; doi:10.1155/crip/9945177)
Supplement: Supplementary file 1 — Supporting Information Additional supporting information can be found online in the Supporting Information section. [file CRIP-2026-9945177-s001.docx]

| **Topic** | **Item** | **Checklist item description** | **Reported on Line** |
| --- | --- | --- | --- |
| **Title** | **1** | The diagnosis or intervention of primary focus followed by the words “case report” | Page 1, Line 1 |
| **Key Words** | **2** | 2 to 5 key words that identify diagnoses or interventions in this case report, including "case report" | Page 1, Line 27 |
| **Abstract** | **3a** | Introduction: What is unique about this case and what does it add to the scientific literature? | Page 1, Line 22-24 |
| **(no references)** | **3b** | Main symptoms and/or important clinical findings | Page 1, Line 22-25 |
|  | **3c** | The main diagnoses, therapeutic interventions, and outcomes . . . . . . . . . . . . . . . . . . . . . . . . . . . . . . . . . . . . . . . . . . . | Page 1, Line 25-26 |
|  | **3d** | Conclusion—What is the main “take-away” lesson(s) from this case? | Page 1, Line 19-22 |
| **Introduction** | **4** | One or two paragraphs summarizing why this case is unique (**may include references**) | Page 1, Line 19-22  Page 4, Line 132-164 |
| **Patient Information** | **5a** | De-identified patient specific information | Page 1, Line 38 |
|  | **5b** | Primary concerns and symptoms of the patient | Page 1, Line 38-41 |
|  | **5c** | Medical, family, and psycho-social history including relevant genetic information | Page 1, Line 41-43 |
|  | **5d** | Relevant past interventions with outcomes | Page 1, Line 41-43 |
| **Clinical Findings** | **6** | Describe significant physical examination (PE) and important clinical findings | Page 1-2, Line 44-46 |
| **Timeline** | **7** | Historical and current information from this episode of care organized as a timeline | N/A |
| **Diagnostic** | **8a** | Diagnostic testing (such as PE, laboratory testing, imaging, surveys) | Page 1, Line 24-25 |
| **Assessment** | **8b** | Diagnostic challenges (such as access to testing, financial, or cultural) | N/A |
|  | **8c** | Diagnosis (including other diagnoses considered) | Page 2-3, Line 86-99 |
|  | **8d** | Prognosis (such as staging in oncology) where applicable | N/A |
| **Therapeutic** | **9a** | Types of therapeutic intervention (such as pharmacologic, surgical, preventive, self-care) | Page 2, Line 47-49 |
| **Intervention** | **9b** | Administration of therapeutic intervention (such as dosage, strength, duration) | Page 2, Line 47-49 |
|  | **9c** | Changes in therapeutic intervention (with rationale) | Page 2, Line 47-49 |
| **Follow-up and** | **10a** | Clinician and patient-assessed outcomes (if available) | Page 3-4, Line 127-129 |
| **Outcomes** | **10b** | Important follow-up diagnostic and other test results | N/A |
|  | **10c** | Intervention adherence and tolerability (How was this assessed?) | N/A (Not collected) |
|  | **10d** | Adverse and unanticipated events | N/A |
| **Discussion** | **11a** | A scientific discussion of the strengths AND limitations associated with this case report | Page 2, Line 52-60 (Limited number of published cases for review) |
|  | **11b** | Discussion of the relevant medical literature **with references** | Page 2-3, Line 55-121 |
|  | **11c** | The scientific rationale for any conclusions (including assessment of possible causes) | Page 2, Line 71-83 |
|  | **11d** | The primary “take-away” lessons of this case report (without references) in a one paragraph conclusion | Page 2-4, Line 84-129 |
| **Patient Perspective** | **12** | The patient should share their perspective in one to two paragraphs on the treatment(s) they received | 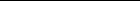N/A |
| **Informed Consent** | **13** | Did the patient give informed consent? Please provide if requested | **Yes √** 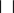 **No** 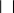 |
